# Diagnosis and treatment of gastric hamartomatous inverted polyp (GHIP) by endoscopic submucosal dissection: A case report

**DOI:** 10.1097/MD.0000000000033443

**Published:** 2023-03-31

**Authors:** Yi-Ping Han, Cong-Cong Min, Yu-Bei Li, Yun-Qing Chen, Hua Liu, Zi-Bin Tian, Xiao-Yan Yin

**Affiliations:** a Department of Gastroenterology, The Affiliated Hospital of Qingdao University, Qingdao, Shandong Province, China; b Department of Pathology, The Affiliated Hospital of Qingdao University, Qingdao, Shandong Province, China.

**Keywords:** case report, endoscopic performance, endoscopic submucosal dissection, gastric hamartomatous inverted polyp, pathological characteristics

## Abstract

Gastric hamartomatous inverted polyps (GHIP) is not a common disease, and it has rarely been reported in the literature. Preoperative diagnosis is difficult due to the deep position and surface covered with normal gastric mucosa. However, with the progress of endoscopic technology, endoscopic submucosal dissection (ESD) can play a crucial role in the diagnosis and treatment of GHIP.

**Patient concerns:**

A 61-year-old Chinese man underwent gastroscopy due to abdominal pain 2 months prior that revealed chronic superficial nonatrophic gastritis with erosion and a submucosal tumor in the gastric body (an ultrasound gastroscopy was recommended). Therefore, he was admitted to our hospital for further diagnosis and treatment.

**Diagnoses:**

A hemispherical submucosal tumor was found in the middle segment of the stomach, with a size of approximately 30 mm × 35 mm and a smooth surface without central ulceration or mucosal bridge formation. Ultrasound gastroscopy showed that the lesion was a hypoechoic mass with uniform internal echo originating from the muscularis propria.

**Interventions:**

The tumor was completely removed by using ESD. The postoperative pathological results indicated a monocystic structure in the submucosa that was not connected with the surface mucosa. The surface of the cyst was covered with foveolar cells and mucous-neck cells (part of which had low-grade intraepithelial neoplasia), and GHIP was considered to be diagnosed.

**Outcomes:**

According to the abovementioned endoscopic and pathological features, the patient was finally diagnosed with GHIP. The patient was successfully discharged after surgery and received regular follow-up observations.

**Lessons:**

GHIP is located in the submucosa layer and has the potential risk of malignant transformation. However, it is not easy to diagnose by using gastroscopy and ultrasound gastroscopy. ESD can obtain complete specimens, which contributes to the diagnosis and treatment of GHIP.

## 1. Introduction

Gastric hamartomatous inverted polyp (GHIP) consists of supramucosal and submucosal polyps with gastric gland hyperplasia and submucosal cystic dilatation.^[1]^ Due to the fact that the main lesion is located in the submucosa or muscularis mucosa, it is difficult to accurately diagnose GHIP by using preoperative biopsy alone.^[2]^ The diagnosis still depends on postoperative pathology, but a clear understanding of endoscopic findings is helpful for our initial screening. Although relatively uncommon, GHIP has been occasionally reported to contain gastric cancer.^[3]^ En bloc removal is recommended in lesions > 2 cm due to the associated malignant risk (up to 20% risk of malignancy).^[4]^ Herein, we report of a case of GHIP that was successfully diagnosed and treated by using endoscopic submucosal dissection (ESD) and discuss its clinicopathological features, diagnosis and treatment to improve the understanding of GHIP.

## 2. Case history

**Figure 1.**
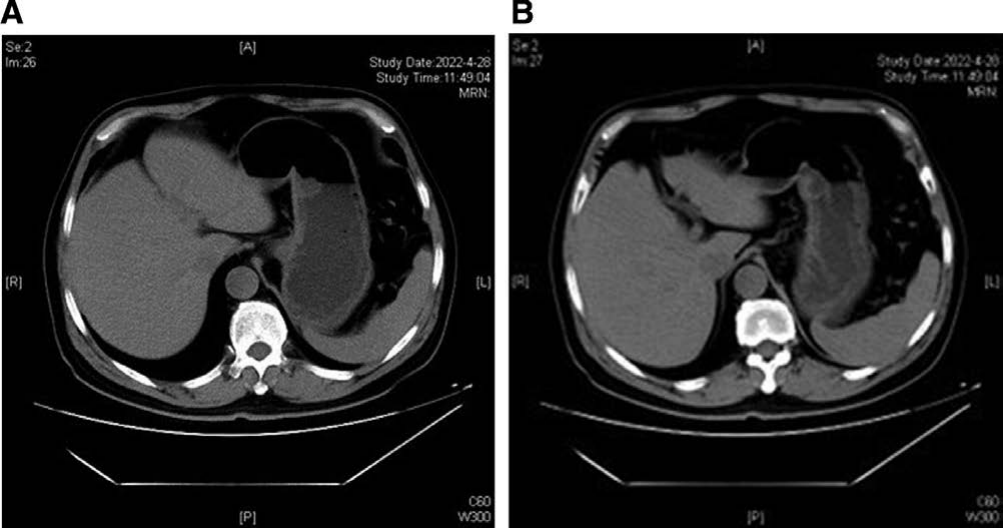
CT findings. (A and B) Abdominal CT showed a round low-density shadow in the small curvature of the stomach. CT = computed tomography.

**Figure 2.**
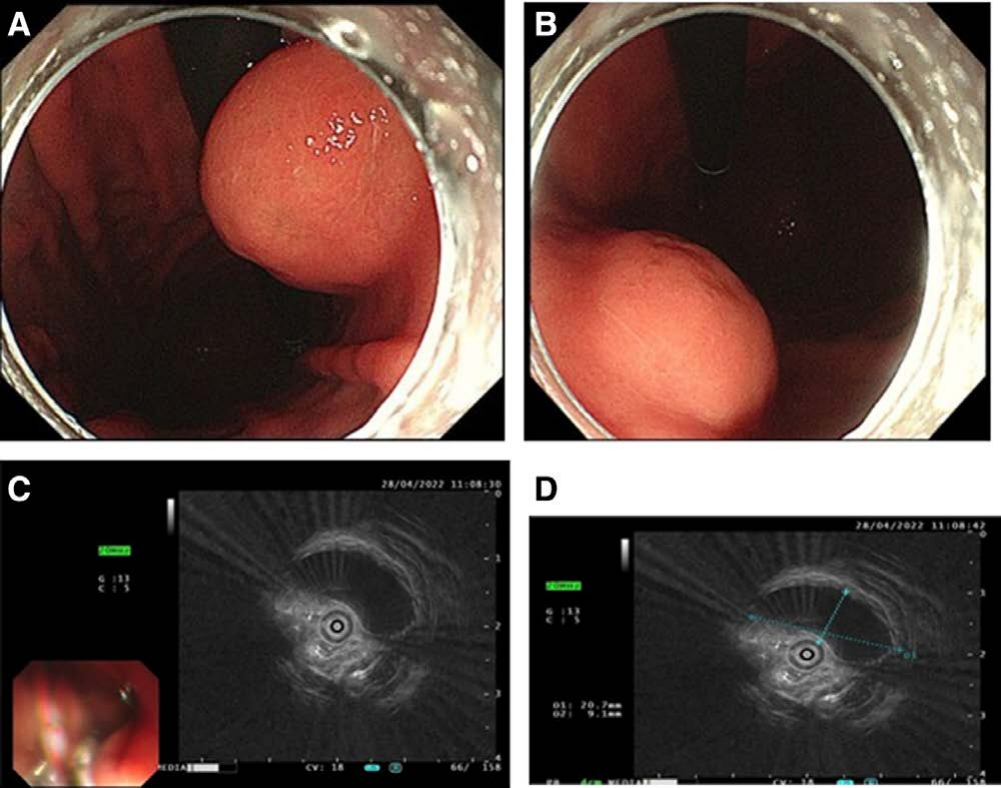
Endoscopic findings. (A and B) A hemispherical smooth submucosal mass with a central depression was observed on white light endoscopy. (C and D) Endoscopic ultrasonography showed a hypoechoic mass with uniform internal echo originating from the muscularis propria layer.

**Figure 3.**
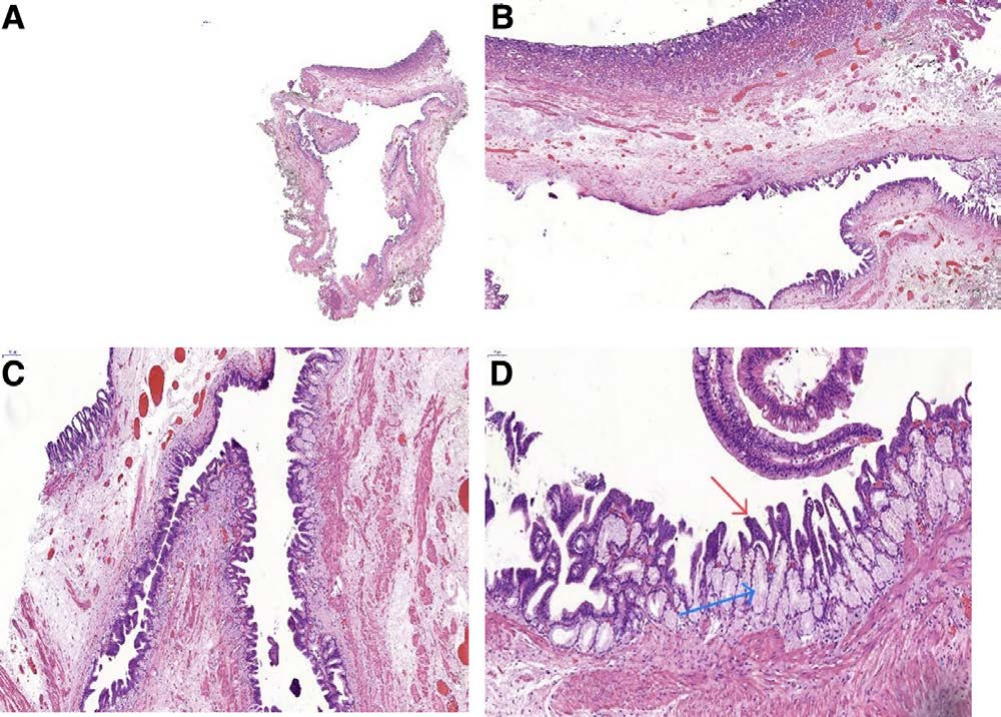
Endoscopic submucosal dissection. The flushing knife 2.0BT was used to cut along the margin to expose the submucosal white tumor (A–D), the base of which was connected to the muscular layer and completely peeled along the margin of the tumor (E–G).

**Figure 4.**
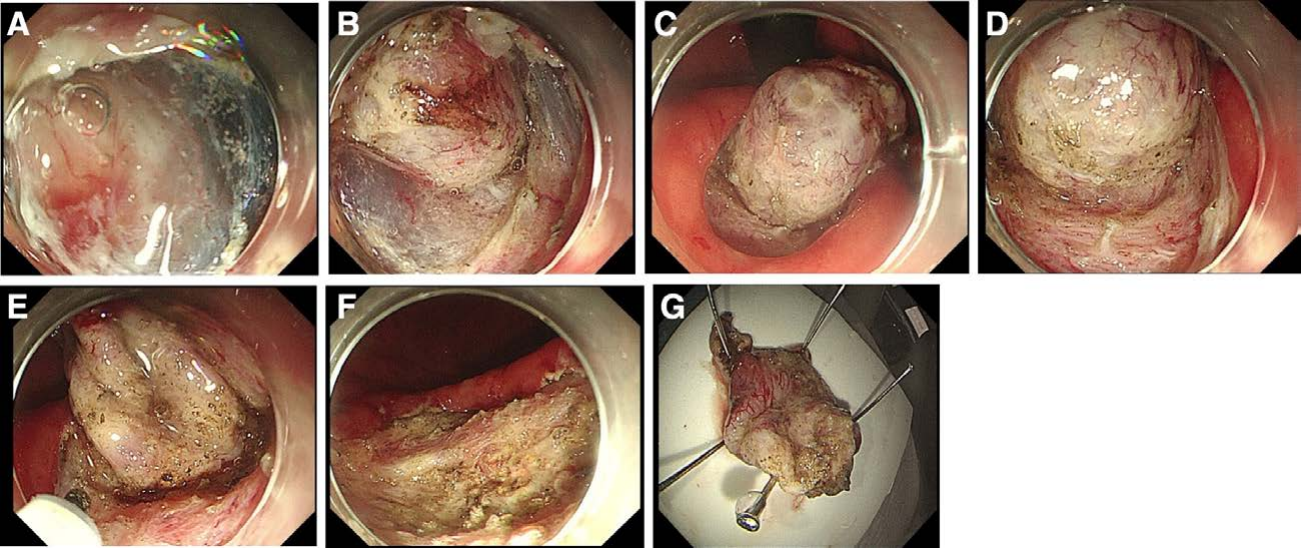
Pathology findings. (A and B) A single cystic structure in the submucosa that was not connected with the surface mucosa. (C and D) the surface of the cyst was covered with foveolar cells and mucinous neck cells, with low-grade intraepithelial neoplasia in some areas (red arrows are foveolar epithelium with low-grade intraepithelial neoplasia; the blue arrow is the mucus gland, which was composed of the constituent mucus cells). (A) Magnification 12 ×, (B) magnification 20 ×, (C) magnification 50 ×, and (D) magnification 100 ×.

A 61-year-old Chinese man underwent gastroscopy due to abdominal pain 2 months prior that revealed chronic superficial nonatrophic gastritis with erosion and a submucosal tumor (SMT) in the gastric body (an ultrasound gastroscopy was recommended). Therefore, he was admitted to our hospital for further diagnosis and treatment. The patient was in good health with no history of surgery or drug allergy. His personal and family history was unremarkable. Physical examination and laboratory data on admission showed no signs of abnormality. Abdominal computed tomography (Fig. 1A and B) and gastroscopy (Fig. 2A and B) showed tumors in the stomach in the lesser curvature of the stomach. In Ultrasound gastroscopy, a hypoechoic mass was observed in the lesser curvature of the gastric body, with uniform internal echo, and it was found to be originating from the muscularis propria, which was considered a possible leiomyoma (Fig. 2C and D). Because it was difficult to make a definitive diagnosis based on these findings, we considered that ESD was necessary to make an accurate diagnosis and select optimal treatment. Subsequently, ESD achieved complete resection of the tumor with 30 × 35 mm in diameter (Fig. 3). Histologically, the tumor was characterized by a monocystic structure in the submucosa, which was not connected with the surface mucosa. The surface of the cyst was covered with foveolar cells and mucous-neck cells, and it was accompanied by low-grade intraepithelial neoplasia (Fig. 4A–D). Immunohistochemistry: CKpan (+), HER2 (0), Syn (−), CgA (−), SMA showed continuous mucosal muscle, as well as S100 (−), CD31 and D2-40 (−), Ki-67 (+, approximately 5%). According to the endoscopic performance and histopathologic characteristics, gastric hamartomata’s inverted polyps were considered for the diagnosis. The patient’s operation was successful and without any postoperative complications.

## 3. Discussion

GHIP is described as gastric lesions of familial colonic polyposis, accounting for <1% of all gastric polyps.^[1]^ GHIP is usually asymptomatic, and some patients may present with epig abdominal discomfort without characteristic presentations. Occasionally, it may present with intestinal obstruction or anemia secondary to chronic blood loss.^[5,6]^ It has also been found that the disease is often associated with deep cystic gastritis.^[7]^ In our case, the patient presented with no specific clinical manifestations (only abdominal pain).

Although GHIP is considered a benign lesion, it has been reported that it is often associated with gastric adenocarcinoma or gastric mucosal epithelial dysplasia.^[8]^ At present, the exact association between gastric cancer and GHIP is still controversial. Although it is rare, occasional gastric cancer has been reported in GHIP.^[3,7,9]^ Approximately 20% of GHIPs were found to be coexisted with precancerous or cancerous areas.^[10,11]^ Due to the fact that GHIP often presents as SMT-like lesions covering normal gastric mucosa, it is difficult to diagnose GHIP carcinogenesis via white light endoscopy and endoscopic ultrasonography (EUS) alone. This patient showed low-grade intraepithelial neoplasia in some areas of postoperative pathology, thus suggesting that GHIP may be associated with the occurrence of gastric cancer; moreover, there is a risk of further development of gastric cancer.

GHIP is thought to occur as a result of infiltration of the mucosa through the muscularis, mucosal crevices or defects caused by repeated erosion.^[12]^ GHIP is usually reported as an isolated submucosal mass on endoscopy.^[5,13]^ Endoscopic examination of a case reported by Dohi showed that the lesion was covered by a normal mucous membrane with a distinct irregular depression at the top.^[14]^ The case reported by Mogi also presented under endoscopy as a submucosal bulge covered by normal gastric mucosa with a red top, and biopsy specimens taken from the red area showed inflammation of the gastric mucosa with hyperplasia and slightly distorted fossa glands.^[15]^ In the case by Takuma Okamura, magnification endoscopy and narrow-band imaging revealed a red area around the depression, as well as an uneven arrangement of irregular villi and pits, with no clear dividing line.^[9]^ Therefore, it is difficult to diagnose GHIP by using endoscopy alone. The findings on endoscopic ultrasonography are usually heterogeneous tumors with cystic spots. Endoscopic ultrasonography of the case reported by Takuma Okamura revealed a nonuniform tumor and cystic area, which was located in the 3rd layer.^[9]^ Moreover, the case reported by Moyu Dohi showed a heterogeneous tumor from the second layer with small cystic hypoechoic spots by ultrasound endoscopy.^[14]^ Suguru Hayase et al^[1]^ reported that GHIP cases showed cystic speckled heterogeneous tumors in EUS, but their location in the gastric parietal layer was unclear. He also showed that other features of EUS imaging have been reported, such as diffuse hyperechoic masses in the submucosa and multicompartment anechoic zones in the 3rd layer. Therefore, we cannot diagnose or exclude GHIP according to which layer the tumor is located, and we can only make a preliminary suspicion of the lesion by using the abovementioned endoscopic ultrasound findings. In this case, gastroscopy showed a 30 mm × 35 mm hemispherical submucosal mass with a smooth surface and central depression without ulceration in the lesser curvature of the gastric body (with the use of biopsy forceps soft to the touch) (Fig. 2A and B). Endoscopic ultrasound revealed a hypoechoic mass from the muscularis propria with homogeneous internal echoes (Fig. 2C and D), which was different from data from previously reported cases.

The pathological definition of GHIP is cystic dilated hyperplastic pseudo pyloric gland hyperplasia and smooth muscle located in the submucosa, with the proliferation of smooth muscle bundles producing branches.^[16]^ Histologically, the tumor is characterized by submucosal gland hyperplasia and cystic structure. The surface of the polyp is gastric fundus gland type or pyloric gland type gastric mucosa. In addition, the glandular structure consists of various types of lining cells, including pyloric or mucus neck cells, mucous (foolar) on the surface of cells, and the cell wall. Submucosal glands or cystic components are connected with the overlying gastric mucosa through mucosal muscularis defects.^[1]^ But the pathological findings of previously reported cases are usually polycystic structures. ^[2,5,17]^ In our case from histological aspects, the tumor was characterized by a single cystic structure in the submucosa that was not connected to the surface mucosa. The cyst surface was covered with foveolar cells and mucinous neck cells, and there was low-grade intraepithelial neoplasia in some areas (Fig. 4).

The diagnosis and treatment of GHIP still demonstrate important problems. Data from the previous literature have described that because the lesions are located in the submucosa or muscularis mucosa, as well as the fact that endoscopy and endoscopic ultrasonography lack specific manifestations, the diagnosis mainly relies on pathological diagnosis. However, biopsy is difficult; therefore, it is not easy to make a diagnosis before resection.^[1,2,14]^ The diagnosis of GHIP is based on the pathological characteristics of the tumor, including fibroblasts, smooth muscle proliferation, neural components, vascular forming tissue, glandular hyperplasia and cyst gland expansion, among other characteristics.^[18]^ Diagnostic resection is currently used in many cases of GHIP. At present, the commonly used treatment methods for GHIP include conventional polypectomy, endoscopic submucosal dissection, laparoscopic wedge resection and endoscopic segmental mucosal resection. ^[6,17]^ The treatment mainly depends on the characteristics and size of the tumor. There are 2 types of hamartomatous inverted polyps: the SMT type (as was observed in the present case), which does not have a stalk, and the polyp type, which has a stalk.^[2]^ Previous studies have reported that all pedicled polypoid types were resected by endoscopy, and all sessile SMT types were surgically resected due to incomplete endoscopic resection in the previous reports.^[1,5,14]^ Later studies have shown that, with the development of endoscopic technology, endoscopic resection was also feasible for the SMT type. Endoscopic resection is comparable to traditional surgery in many aspects and has the advantages of inducing less trauma and decreased economic burdens.^[2]^ Conventional endoscopic mucosal resection can be used for monolithic resection of SMT lesions when the lesion diameter is less than or equal to 10 mm.^[19]^ For SMT type lesions with a diameter of 10 mm or greater, ESD is chosen to prevent occurrences of incomplete resection.^[1,14]^ In this case, the 30 mm × 35 mm SMT-type lesion was successfully removed by using the ESD method and submitted for pathological examination, and GHIP was finally diagnosed.

## 4. Conclusion

GHIP accounts for <1% of all gastric polyps, with no characteristic clinical manifestations, and it can occasionally cause intestinal obstruction or chronic anemia. The exact association between gastric cancer and GHIP is still controversial, and approximately 20% of GHIP coexists with precancerous or cancerous regions. The diagnosis of GHIP depends on the postoperative pathological results of diagnostic resection, which usually show cystic, dilated and hypertrophic pseudopyloric gland hyperplasia. In terms of treatment, with the continuous progress of endoscopic technology, endoscopic therapy may become the first choice for the treatment of this lesion.

## Acknowledgments

We thank all of the authors who contributed to this article.

## Author contributions

**Conceptualization:** Yubei Li, Yunqing Chen, Hua Liu, Zibin Tian, Xiaoyan Yin.

**Formal analysis:** Yiping Han, Congcong Min, Yubei Li, Yunqing Chen, Hua Liu, Zibin Tian, Xiaoyan Yin.

**Funding acquisition:** Congcong Min.

**Investigation:** Yiping Han, Yubei Li, Yunqing Chen, Xiaoyan Yin.

**Project administration:** Yiping Han, Yubei Li, Hua Liu, Zibin Tian, Xiaoyan Yin.

**Resources:** Yunqing Chen, Hua Liu, Xiaoyan Yin.

**Supervision:** Congcong Min, Yunqing Chen, Hua Liu, Zibin Tian, Xiaoyan Yin.

**Validation:** Yiping Han, Congcong Min, Yunqing Chen, Hua Liu, Zibin Tian.

**Visualization:** Yiping Han, Yubei Li.

**Writing – original draft:** Yiping Han, Congcong Min, Yubei Li, Zibin Tian, Xiaoyan Yin.

**Writing – review & editing:** Yiping Han, Congcong Min, Yubei Li, Hua Liu, Zibin Tian, Xiaoyan Yin.
